# Allelic variants between mouse substrains BALB/cJ and BALB/cByJ influence mononuclear cardiomyocyte composition and cardiomyocyte nuclear ploidy

**DOI:** 10.1038/s41598-020-64621-0

**Published:** 2020-05-05

**Authors:** Peiheng Gan, Michaela Patterson, Hirofumi Watanabe, Kristy Wang, Reilly A. Edmonds, Laura G. Reinholdt, Henry M. Sucov

**Affiliations:** 1 Department of Regenerative Medicine and Cell Biology, Medical University of South Carolina, Charleston, SC USA; 2 Department of Stem Cell Biology and Regenerative Medicine, University of Southern California Keck School of Medicine, Los Angeles, CA USA; 3 Department of Cell Biology, Neurobiology and Anatomy, and Cardiovascular Center, Medical College of Wisconsin, Milwaukee, WI USA; 4 The Jackson Laboratory for Mammalian Genetics, Bar Harbor, ME USA; 5 Department of Medicine, Division of Cardiology, Medical University of South Carolina, Charleston, SC USA

**Keywords:** Developmental biology, Cardiology

## Abstract

Most mouse cardiomyocytes (CMs) become multinucleated shortly after birth via endoreplication and interrupted mitosis, which persists through adulthood. The very closely related inbred mouse strains BALB/cJ and BALB/cByJ differ substantially (6.6% vs. 14.3%) in adult mononuclear CM level. This difference is the likely outcome of a single X-linked polymorphic gene that functions in a CM-nonautonomous manner, and for which the BALB/cByJ allele is recessive to that of BALB/cJ. From whole exome sequence we identified two new X-linked protein coding variants that arose de novo in BALB/cByJ, in the genes Gdi1 (R276C) and Irs4 (L683F), but show that neither affects mononuclear CM level individually. No BALB/cJ-specific X-linked protein coding variants were found, implicating instead a variant that influences gene expression rather than encoded protein function. A substantially higher percentage of mononuclear CMs in BALB/cByJ are tetraploid (66.7% vs. 37.6% in BALB/cJ), such that the overall level of mononuclear diploid CMs between the two strains is similar. The difference in nuclear ploidy is the likely result of an autosomal polymorphism, for which the BALB/cByJ allele is recessive to that of BALB/cJ. The X-linked and autosomal genes independently influence mitosis such that their phenotypic consequences can be combined or segregated by appropriate breeding, implying distinct functions in karyokinesis and cytokinesis.

## Introduction

In mammals, only a small percentage of adult ventricular cardiomyocytes (CMs) are diploid (i.e., have a single 2n nucleus (n is the haploid chromosomal content), also called mononuclear diploid or abbreviated as 1 × 2n) (reviewed in^1,2^). Through a process known as endoreplication, CMs enter cell cycle and progress through S-phase DNA replication, but then fail to complete mitosis. The first iteration of endoreplication results in CMs having either a single tetraploid nucleus (1 × 4n) or two diploid nuclei (2 × 2n), depending on whether mitosis was interrupted before completion of karyokinesis or of cytokinesis, respectively. Both states are considered to be polyploid, as both have four chromosome sets per cell. In rats and mice^3–5^ and several large animals and likely also humans^6^, the peak of CM endoreplication occurs shortly after birth, whereas in lambs it likely occurs in late gestation^7^. Subsequent reiterations of this process, as occur naturally in postnatal life and in the aftermath of injury or disease, can result in CMs with higher numbers of nuclei, higher numbers of genomes per nucleus, or both. Polyploidy is not unique to CMs, as certain other cell types also have polyploid subpopulations. The liver has been extensively studied in this regard; in adult mice, approximately 90% of hepatocytes are polyploid^8^.

The relevance of polyploidy to cell biology and organ physiology has been the subject of speculation for decades. Polyploid CMs are larger than diploid CMs; candidate roles other than size that may distinguish diploid and polyploid CMs include regenerative capacity, sensitivity to oxidative stress, contractility, gene expression, metabolism, and others^1,2^. Our approach has been to identify genetically-encoded variation in the extent of polyploidy as a first step towards defining how polyploidy occurs and its biological relevance. We proposed and then showed in mice^9^ that the frequency of diploid CMs in the normal adult mammalian ventricle is not a fixed trait, but rather exhibits substantial natural variation based on the combined effects of a number of alleles that are polymorphic between individuals. In mice, most polyploid CMs are binucleated with two diploid nuclei (2 × 2n). Therefore, in this prior analysis, we first surveyed a large number of inbred mouse strains for the percentage of mononuclear CMs, and then measured nuclear ploidy specifically within the mononuclear CM subset only for selected strains. We found 7-fold variation in the percentage of mononuclear CMs (range 2.3–17.0%), but less than 2-fold variation in the percentage of diploid nuclei in mononuclear CMs (range 40–70%). Thus, we used the simple measurement of mononuclear CM level as a surrogate for diploid CM level. Because other variables (housing, age, sex, etc.) were controlled, genetic variation is the most likely explanation for the observed phenotypic variation between strains. As a demonstration of this principle, by genome-wide association we identified a natural loss-of-function variant in the gene Tnni3k in many inbred strains that have a high level of mononuclear CMs, and confirmed in a controlled C57BL/6J strain background (which normally carries the functional wild-type Tnni3k allele) that mutation of this gene resulted in a 2–3-fold increase in the percentage of adult mononuclear CMs and in the percentage of diploid CMs^9,10^. Clearly, many genes in mice in addition to Tnni3k have natural variants that also influence the frequency of this CM population. The identification of these genes and their natural variants, and how their products function in cell cycle control, karyokinesis, and cytokinesis, is of significance for reaching a better understanding of the causes and consequences of CM polyploidy and how this influences adult heart biology.

In our survey of mononuclear CM content across inbred mouse strains^9^, we observed a surprising discrepancy between the sister strains BALB/cJ (5.9% mononuclear CM) and BALB/cByJ (14.0%) (both values are slightly higher in the reevaluation reported in the present study). The BALB/cJ level was very close to the median (6.1%) among the 120 inbred mouse strains evaluated, whereas BALB/cByJ was quite high (rank 116 of 120). The difference in mononuclear CM content between BALB/cJ and BALB/cByJ is therefore substantial. These two strains originated from an already-inbred Balb stock, were separated in 1935, and have since been kept in reproductive isolation^11^. Thus, phenotypic differences between these substrains today arose by spontaneous mutation during the past 85 years and then became fixed by inbreeding. Because they are so closely related, it is possible that one or a small number of mutations unique to one or the other strain might account for this difference. Because of the prediction that the relevant variant(s) arose uniquely and recently within only one of the two sublines and is therefore not widely distributed over many inbred strains, genome-wide association as in our previous analysis^9^ would not be expected to detect its presence regardless of the magnitude of its effect.

The goal of this study was to explore the genetic basis of the divergence in CM composition between BALB/cJ and BALB/cByJ, and the implications of this divergence for the general subject of diploid and polyploid CMs in mice.

## Results

### Analysis of BALB/cJ and BALB/cByJ parental strains

**Figure 1:**
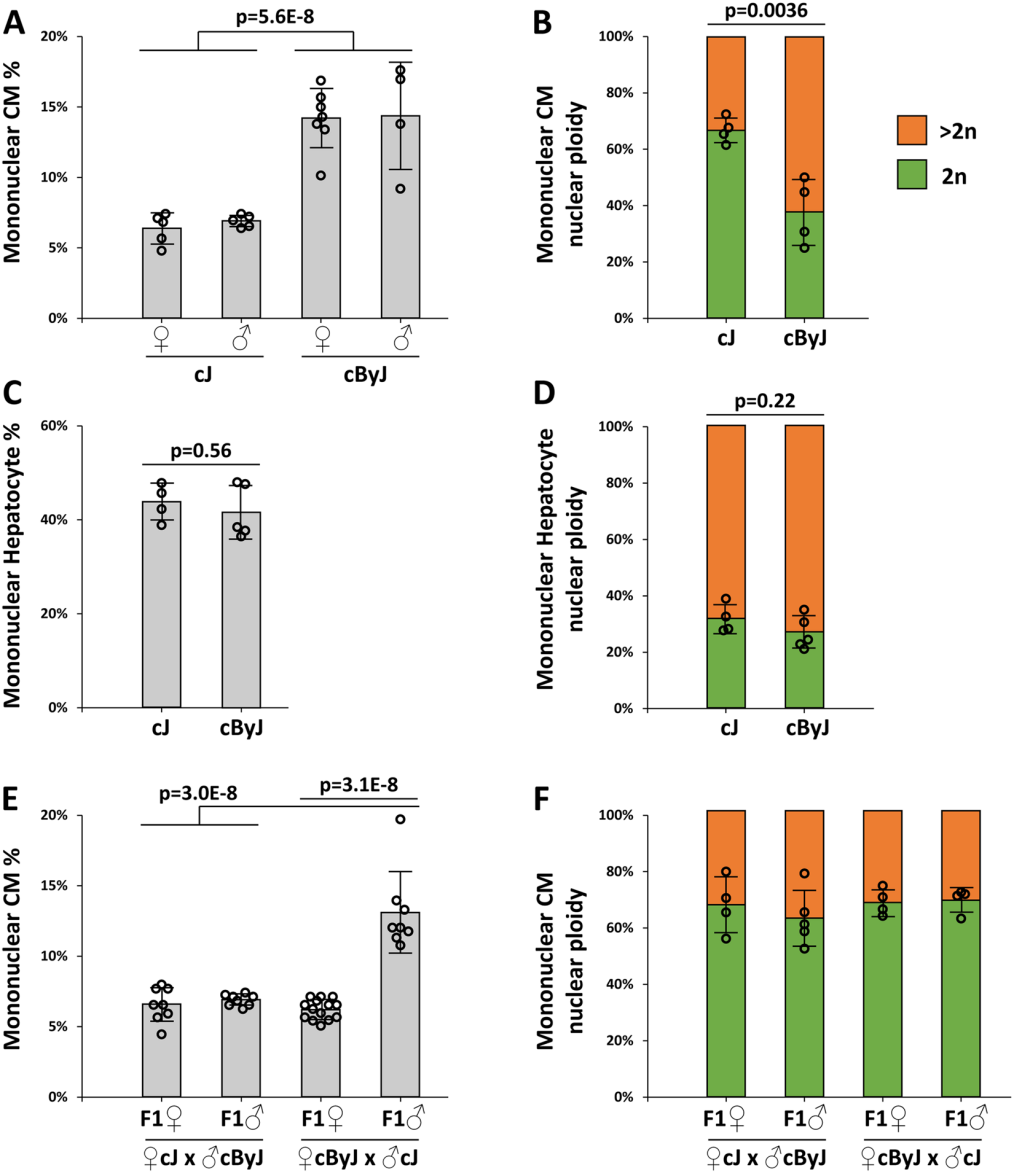
CM nucleation and nuclear ploidy. (**A**) Evaluation of CM mononuclear percentage of male and female mice of the BALB/cJ (abbreviated as cJ) and BALB/cByJ (abbreviated as cByJ) parental strains. 3 data points for BALB/cJ females and 3 data points for BALB/cByJ females are from a prior analysis^9^, all others were newly generated in this analysis. (**B**) Evaluation of the nuclear ploidy specifically of the mononuclear CM subpopulation of the parental strains. The data points graphed are measurements of the percentages of diploid nuclei in individual mice. (**C**) Evaluation of hepatocyte mononuclear percentage in the two substrains. (**D**) Evaluation of the nuclear ploidy specifically of the mononuclear hepatocyte subpopulation. (**E**) Evaluation of CM mononuclear percentage of male and female F1 mice bred from crosses of the two parental strains. (**F**) Evaluation of nuclear ploidy of the mononuclear CM subpopulation of F1 mice. Primary data for panels B and F are in Supplementary Fig. S1 and S4. Error bars in all panels are standard deviation.

In order to control sex as a variable, our original survey of mononuclear CM content across inbred mouse strains^9^ evaluated only female mice. We first addressed whether the striking difference in mononuclear CM content between BALB/cJ and BALB/cByJ adult females was also true in males. Indeed, for both substrains, males demonstrated the same mononuclear CM level as females (Fig. 1A). Our past work with C57BL/6J mice also showed no sex difference in this parameter^9,10^. Combining male and females together, our revised calculation of ventricular mononuclear CM frequency is 6.6% for BALB/cJ, and 14.3% for BALB/cByJ, which is more than a 2-fold difference. Although the ventricular CM populations of BALB/cJ and BALB/cByJ are both overwhelmingly multinucleated (93.4% and 85.7%, respectively), the>2-fold elevation in mononuclear CMs in BALB/cByJ is a substantial difference and is of a magnitude similar to Tnni3k gene mutation^9^.

In general, because most adult mouse CMs are binucleated, many studies in the literature have evaluated CM nuclear number but not also nuclear ploidy, under the assumption that a relatively consistent percentage of mononuclear CMs are mononuclear diploid and mononuclear tetraploid. As noted above, our initial large-scale survey of inbred mouse lines also scored first for the frequency of mono- vs. multi-nucleated CMs, and conducted direct measurement of nuclear ploidy only for specific selected strains. To address this explicitly for BALB/cJ and BALB/cByJ, we measured nuclear ploidy by quantification of nuclear DAPI signal intensity (normalized to the signal intensity from endothelial cells (Supplementary Fig. S1), which are assumed to be diploid unless in the process of mitosis), as we^10^ and others have used in the past. Surprisingly, we found a striking distinction in mononuclear CM nuclear ploidy between the two strains (Fig. 1B, Supplementary Fig. S1): in BALB/cJ mice, 66.7% of the mononuclear CM nuclei were diploid, compared to only 37.6% in BALB/cByJ. While these numbers are consistent with the 40–70% range previously observed when comparing other inbred mouse strains^9^, it was unexpected to find this magnitude of difference in such closely related substrains. The combination of mononuclear CM percentage (Fig. 1A) and mononuclear CM nuclear ploidy (Fig. 1B) allows calculation of the level of diploid ventricular CMs in the two substrains: 4.4% for BALB/cJ, and 5.4% for BALB/cByJ. Thus, the two BALB strains differ in mononuclear CM percentage in one direction, and differ in mononuclear CM nuclear ploidy in the opposite direction, such that their levels of diploid CMs are similar.

**Figure 2:**
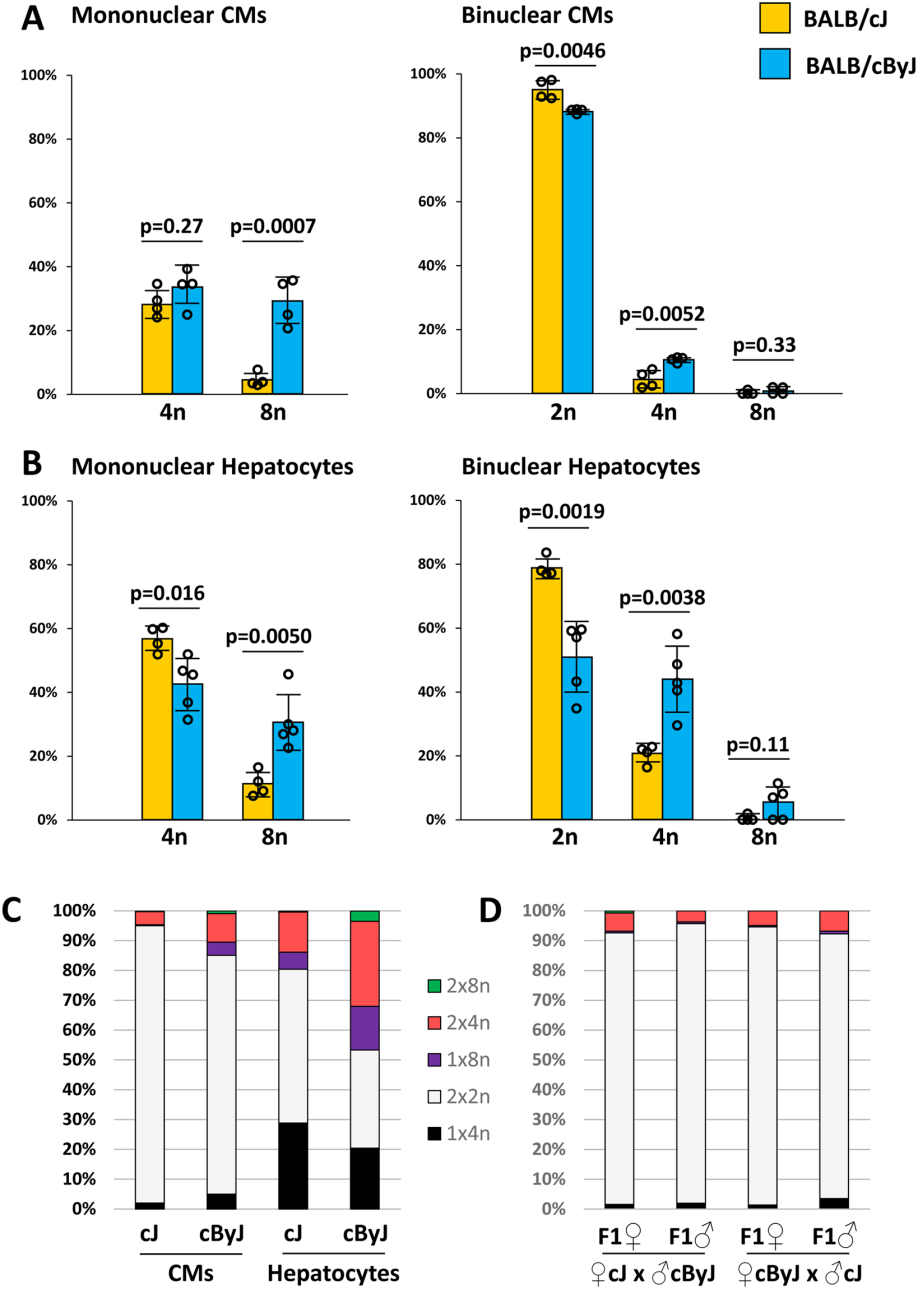
Polyploidy variation between BALB/cJ and BALB/cByJ. (**A**) CM analysis. Within the mononuclear CM and binuclear CM subgroups, the percentages of nuclei of the indicated ploidy (2n, diploid; 4n, tetraploid; 8n, octoploid) are shown. The 2n (diploid) percentage of mononuclear CMs is shown in Fig. 1B and not repeated here. Data were calculated in this manner because a disproportionately greater number of mononuclear CM nuclei were evaluated relative to their frequency in the heart. Error bars represent standard deviation. (**B**) Hepatocyte analysis. Same evaluation as for CMs; the 2n (diploid) percentage of mononuclear hepatocytes is shown in Fig. 1D and not repeated here. (**C**) Distribution of different polyploid subtypes for both CMs and hepatocytes, multiplying the data from panels A and B with the percentage of mononuclear and binuclear cells shown in Fig. 1A,C. 1 × 4n and 2 × 2n cells are both tetraploid; 1 × 8n and 2 × 4n cells are both octoploid. A small number of 2 × 8n cells were observed; there were too few cells with other types of polyploidy to show on this chart. (**D**) Analysis of CM polyploidy levels in F1 mice derived from crosses of BALB/cJ and BALB/cByJ parents in both directions; same color scheme as in panel C.

We also isolated hepatocytes (Supplementary Fig. S2) and evaluated their polyploidy in BALB/cJ and BALB/cByJ mice. In both strains, slightly less than half of hepatocytes were mononuclear (43.7% for BALB/cJ; 42.6% for BALB/cByJ; Fig. 1C), and in both strains approximately 30% of the mononuclear hepatocytes had diploid nuclei (31.9% for BALB/cJ; 26.8% for BALB/cByJ; Fig. 1D). Thus, the prominent differences between these strains in mononuclear CM level and mononuclear CM nuclear ploidy level are not also manifest in mononuclear hepatocytes. Combining these values yielded a calculated level of diploid hepatocytes of 13.6% and 11.4% in BALB/cJ and BALB/cByJ, respectively, which is consistent with prior observations in mice^8^. Interestingly, a closer evaluation revealed that BALB/cByJ had a higher degree of polyploidy in both polyploid CMs and polyploid hepatocytes (Fig. 2). That is, in both cell types, BALB/cByJ had a higher level of octaploid (1 × 8n and 2 × 4n) cells, whereas BALB/cJ had proportionately higher percentage of tetraploid (1 × 4n and 2 × 2n) cells. The spectrum of polyploid CMs in BALB/cJ was similar to C57BL/6J and other mouse strains we have studied in the past^9,10^, whereas that of BALB/cByJ was novel. Tetraploid 2 × 2n cells were still the most common type of polyploid CM in BALB/cByJ. Endothelial cells in both strains were uniformly mononucleated and their nuclear DAPI fluorescence intensity clustered narrowly around a median value (i.e., were diploid) (Supplementary Fig. S1A). Similarly, analysis of bone marrow cells (stromal and hematopoietic combined) showed no binucleated cells and only a modest percentage (identical in the two strains) of polyploid nuclei (Supplementary Fig. S3) that are presumed to be of diploid cells in mitosis and polyploid megakaryocytes.

### Analysis of F1 mice

To gain insight into the inheritance pattern of the variants that distinguish BALB/cJ and BALB/cByJ, we crossed the two parental lines in both directions and evaluated male and female F1 mice in the same manner as above. Variation in mononuclear CM percentage (Fig. 1E) segregated in a binary manner consistent with an X-linked allele, and with the variant in BALB/cByJ being recessive to that in BALB/cJ. That is, the higher mononuclear CM level was only observed in F1 male mice that carried a single X chromosome inherited from their BALB/cByJ mother, and not in F1 female mice regardless of direction of cross. Other explanations, including maternal biology and epigenetic/imprinting inheritance, are inconsistent with these results. It is likely that the relevant gene functions in a CM-nonautonomous manner, as F1 females showed only the lower (BALB/cJ-parental) level and not an intermediate level as would be predicted for a CM-autonomous trait subject to X-inactivation.

Assessment of nuclear ploidy within the mononuclear CM subpopulation of F1 mice revealed an equivalent percentage of diploid and polyploid nuclei (Fig. 1F, Supplementary Fig. S4). This level (64–70% diploid nuclei) is equivalent to that of the parental BALB/cJ strain (66.7%; Fig. 1B). This suggests that difference in nuclear diploid/polyploid ratio between these two substrains is inherited in an autosomal manner, with the BALB/cJ allele being dominant to that from BALB/cByJ. If the relevant gene is autosomal, no conclusion can be made regarding whether gene function is CM-autonomous or nonautonomous. The analysis also reveals that the two traits of mononuclear CM percentage and mononuclear CM nuclear diploidy appear to segregate independently in these crosses. As a result, the level of diploid CMs in F1 male mice derived from BALB/cByJ mothers (13.1% × 69.9% = 9.2%) is substantially higher than in either parental line (4.4% and 5.4%; see above). A 9.2% level of diploid CMs is among the highest levels we have measured among natural inbred strains (e.g., 10.0% in A/J, 9.3% in SWR/J)^9^, and much higher than in C57BL/6J mice (2.5%). The tendency of polyploid CMs to reach higher ploidy levels in BALB/cByJ mice was not seen in the F1 mice (Fig. 2), which is consistent with this feature also being a manifestation of a recessive allele in BALB/cBy. We cannot yet say if this trait is independent of, or related to, either the X-linked allele that influences mononuclear CM percentage or the autosomal allele that influences the mononuclear CM nuclear diploid/polyploid ratio.

We assume in the above that a single polymorphic X-linked gene is responsible for variation in mononuclear CM level, and similarly that a single polymorphic autosomal gene is responsible for variation in the diploid level of mononuclear CM nuclei. It is formally possible that two (or more) genes are involved in each case, although this seems less likely given the relatively recent divergence between the two substrains. Furthermore, because karyokinesis and cytokinesis are both linked to cell cycle, we also note the alternative possibility that a more complex explanation of inheritance that involves interaction between the autosomal and X-linked alleles is possible.

### Identification and evaluation of Gdi1 and Irs4 as candidate genes

We took an informatics approach to derive candidate X-linked genes that might be responsible for mononuclear CM variation between BALB/cJ and BALB/cByJ mice. Because both current strains originated from the same already-inbred stock, we based our approach on the assumption that the relevant variant must have arisen de novo in one or the other line after their segregation in 1935, and therefore would be unlikely to be present in any other inbred strain unless derived from either parental source. Complete genome sequence is available for 36 inbred mouse strains^12^, including BALB/cJ but not BALB/cByJ. Among these fully sequenced strains, we surveyed for all X-linked gene variants in BALB/cJ that have nonsynonymous or premature stop protein coding changes or splice donor/acceptor mutations, but none were unique to BALB/cJ or shared only with SEA/GnJ (which was derived from a cross of BALB/cJ to P/J in the mid-1940s^13^). The BALB/cJ coding variant shared with the fewest other sequenced inbred strains was rs31755951 in the Vgll1 gene (Supplementary Fig. S5). Direct sequencing showed that this variant is also present in BALB/cByJ (Supplementary Fig. S5), indicating that it predates the 1935 segregation of the two. Indeed, strains A, C3H, and BALB/c, which all share the Vgll1 variant, have some common early (pre-1920) ancestry, likely including this portion of the X chromosome. The X chromosome of BALB/cJ in the 159.6–164.04 Mb region is derived from wild mice and is highly divergent from common inbred mouse strains although shared by SEA/GnJ. Within this region, we sequenced four coding region variants in BALB/cJ (and SEA/GnJ) but found all were shared by BALB/cByJ (Supplementary Fig. S5). Thus, genome sequence failed to reveal an X-linked protein coding change in BALB/cJ mice that could account for the divergence in mononuclear CM content with BALB/cByJ mice.

**Figure 3:**
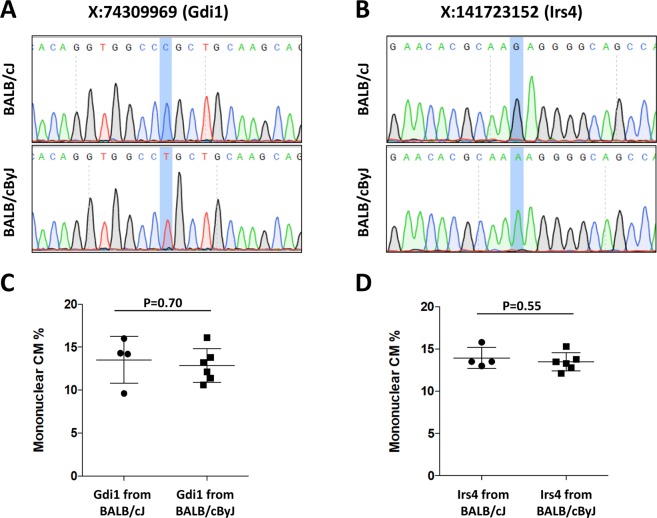
Identification and evaluation of two BALB/cByJ-specific X-linked variants. (**A,B**) Sequence traces of the Gdi1 (A) and Irs4 (B) genes in BALB/cJ and BALB/cByJ mice. (**C,D**) Mononuclear CM percentage in backcrossed F5 male mice grouped by the parental strain origin of their Gdi1 (C) or Irs4 (D) genes.

Alternatively, the relevant mutation might have arisen uniquely in the BALB/cByJ lineage. Whole genome sequence is not available for BALB/cByJ, but we obtained whole exome sequence and filtered for nonsynonymous or functional variants on the X chromosome. We identified two previously unreported polymorphisms (Gdi1, X:74309969 C/T, R276C; Irs4, X:141723152 G/A, L683F) uniquely in BALB/cByJ relative to all other sequenced mouse genomes, including that of BALB/cJ, and confirmed these as being different between BALB/cJ and BALB/cByJ by direct genome sequencing (Fig. 3A,B). We conducted a parallel assessment of whole exome sequence from BALB/cJ mice, although this failed to reveal any new polymorphism not noted above.

To address the candidate role of the Gdi1 and Irs4 alleles, we crossed the two parental lines to generate F1 females, then backcrossed these to BALB/cByJ males for 3 further generations, selecting females at each generation that by meiotic crossover retained only one of the BALB/cJ-specific X-linked alleles (heterozygous to the BALB/cBy allele). F4 females were again crossed to BALB/cByJ males, and F5 males were evaluated for mononuclear CM level. In principle, these F5 males are >90% homozygous for BALB/cByJ alleles over the entire genome except around the individual BALB/cJ variants that were subject to selection. We determined that the strain origin of both variant genes did not alter the mononuclear CM content of the mice (Fig. 3C,D), which was equivalent to the parental BALB/cByJ level. These two variants are thus unlikely to individually explain divergence in this trait between the two strains. Because there are many autosomal variants between the two strains, we did not attempt in this study to evaluate these for their influence on the nuclear ploidy phenotype.

## Discussion

The polyploid nature of almost all adult cardiomyocytes in human, mouse, rat, and several other species has been known for decades, and yet the mechanisms that account for this outcome have remained obscure. Analysis of the natural genetic variation present among inbred mouse strains is one strategy to discover genes that are functionally relevant to any variable mouse trait and therefore to its underlying processes. In this way, by surveying a large number of inbred mouse strains, we previously identified one polymorphic gene (Tnni3k) that influences how many diploid CMs are present^9^. The analysis also uncovered the substantial difference in mononuclear CM level between BALB/cJ and BALB/cByJ that served as the starting point for the present study.

Our analysis of BALB/cJ and BALB/cByJ mice revealed two significant principles. First, the simplifying assumption that the easily measured level of mononuclear CMs in the adult mouse heart is a suitable surrogate for the level of diploid CMs is incorrect, at least in some cases if the mononuclear tetraploid CM population is unusually high or low. As we found here, inclusion of nuclear ploidy measurement essentially erased the substantial difference in mononuclear CMs between the very closely related BALB/cJ and BALB/cByJ substrains. Because differences in nuclear ploidy are based on genetic polymorphisms, this observation is a particularly relevant caution for studies that are conducted on an outbred or mixed strain background, where littermate animals or animals from the same colony would not necessarily serve as appropriate genetic controls.

The second general conclusion of this study is that genes can selectively influence mononuclear CM or CM nuclear ploidy levels in an independent and separable manner. Here, of the two presumptive genes that differ between BALB/cJ and BALB/cByJ, because one gene is X-linked and the other is autosomal, we could easily discern the separable nature of both in the F1 crosses. We interpret differences in nuclear ploidy to indicate effects at the time of karyokinesis, and similarly, genes that influence mononuclear CM level to indicate effects on cytokinesis. Because the machinery and regulation of karyokinesis and cytokinesis are overlapping but also distinct, that there are genes that selectively influence one or the other may not be surprising. Indeed, among mammalian species, although the vast majority of CMs are polyploid, the nature of CM polyploidy varies from being primarily binucleated with two diploid nuclei (2 × 2n) as in mice and rats to primarily mononuclear tetraploid (1 × 4n) in humans^14^. Different inbred mouse strains also vary in mononuclear CM nuclear ploidy^9^. Thus, between species and within a species, and now shown even between very closely related sister strains of mice, there is a genetic basis that distinguishes interruption of endoreplication prior to karyokinesis or prior to completion of cytokinesis as CMs become polyploid.

In retrospect, because our initial survey of 120 mouse strains^9^ only addressed mononuclear CM level, genome-wide association based on strain variation in this parameter might be predicted to yield genes that are selectively relevant to cytokinesis. Indeed, our identification and evaluation of Tnni3k from that study confirmed that this gene primarily impacts mononuclear CM level and not nuclear ploidy, at least when addressed on a controlled C57BL/6J background^9,10^. Statistical significance in genome-wide association is dependent on allele effect size and allele frequency; the latter favors discovery of genes with polymorphic alleles that are widely distributed among the sampled population. For example, the natural Tnni3k variant allele was present in 60 of the 120 strains that we surveyed for mononuclear CM content^9^. For being recent de novo mutations likely present in only a single parental strain, the two (or potentially more) still-unknown BALB variants that influence CM ploidy would not likely have been evidenced in our genome-wide association analysis. The 120 surveyed strains included 8 CXB recombinant inbred lines, which were derived from crosses started in 1959 of BALB/cByJ with C57BL/6ByJ^15^, and also the Sea/GnJ strain, which was derived from a cross begun in the mid-1940s between BALB/cJ and P/J^13^, i.e., both occurred after the 1935 separation of BALB/cJ and BALB/cByJ. However, even with these, the population would still have been underpowered to reveal a statistically significant association. Because BALB/cJ and BALB/cByJ are so closely related, we were able to use a different approach (F1 analysis) to demonstrate that variants in two distinct genes are present, one of which influences mononuclear CM level and one that influences mononuclear CM nuclear ploidy. Such an approach would be not be possible with more distantly related strains because of the complicating influence of numerous additional gene variants that impact either or both features.

While the existence of two variant alleles (one X-linked, one autosomal) is the most parsimonious explanation for the differences between BALB/cJ and BALB/cByJ, we note the possibility that additional genes or a more complex pattern of inheritance might be involved. A definitive conclusion will only be possible once the relevant variants are identified. In the present study, we did not attempt to identify autosomal variants for their role in CM nuclear ploidy, and we were not able to identify the X-linked gene that distinguishes BALB/cJ and BALB/cByJ in mononuclear CM level. Both BALB/cByJ alleles appear to be recessive to their counterparts in BALB/cJ, and thus in principle could be loss-of-function protein coding variants that arose selectively in the former. We identified two new X-linked protein coding variants in BALB/cByJ (in Gdi1 and Irs4) but our data exclude both from being individually relevant to this trait, and analysis of whole exome and full genome sequence of BALB/cJ did not reveal any X-linked protein coding or functional variant that isn’t also shared with BALB/cByJ or among at least several other fully sequenced inbred strains. There are several possible explanations. First, the relevant allele might not be a coding region variant but rather a regulatory variant that influences gene expression. There are too many noncoding variants in BALB/cJ to evaluate these as individual candidates, and we do not have whole genome sequence of BALB/cByJ with which to compare nontranslated regions of the genome. Second, more than one X-linked gene might be involved. In an attempt to specifically address the candidacy of Gdi1 and Irs4 in the phenotype, we segregated the two BALB/cJ alleles by meiotic recombination and backcrossed these individually to BALB/cByJ. However, the two might work together, or either might require an autosomal BALB/cJ variant in order to manifest its effect. Other explanations are possible as well.

Our data reveal that two (or perhaps more) recessive alleles distinguish BALB/cByJ from BALB/cJ mice, one influences mononuclear CM percentage and the other influences CM nuclear ploidy. Curiously, the two influence CM polyploidy in opposite directions: relative to BALB/cJ, BALB/cByJ has a higher number of mononuclear CMs but a higher percentage of these nuclei are polyploid. Thus, the level of diploid CMs between the two substrains is similar (4.4% and 5.4%). This could suggest that an excess of diploid CMs is detrimental such that the two alleles arose together out of necessity. However, because other inbred mouse strains^9^, and F1 male mice derived from BALB/cByJ mothers and BALB/cJ fathers (this study) all have a high diploid CM level without apparent ill effect, we think the occurrence of two variant alleles in BALB mice that both influence CM ploidy is more likely to be a coincidence rather than the outcome of selection.

All cell types, whether diploid or polyploid in the adult, have gone through numerous rounds of mitosis earlier in their lineages. Gene variants that influence either karyokinesis or cytokinesis to result in polyploid cells are clearly not doing so in all circumstances of mitosis, implying their participation in a unique program that influences the outcome specifically in endoreplication. An unexpected observation in this study was that phenotypic variation between BALB/cJ and BALB/cByJ in mononuclear CM level (the X-linked variant) and of mononuclear CM nuclear diploidy (the autosomal variant) were both manifest in CMs but not in hepatocytes (Fig. 1). This suggests that the mechanisms governing cytokinesis and karyokinesis in these two types of endoreplicating cells might be sufficiently distinct from each other to be selectively influenced by variants in these genes. Alternatively, the genes may be selectively expressed in CMs, just as Tnni3k is only expressed in CMs.

A feature that was shared between CMs and hepatocytes was the tendency of both to reach higher levels of polyploidy in the BALB/cByJ background (Fig. 2). This could reflect a tendency to initiate additional rounds of endoreplication, but other explanations are also possible. It is unknown if this is the manifestation of an additional genetic locus in BALB/cByJ, or is in some manner the result of the influence of the X-linked and autosomal loci that are presumptively involved in cytokinesis and karyokinesis, respectively. This behavior was inherited in autosomal manner, with the BALB/cByJ allele(s) being recessive to BALB/cJ.

Although the new BALB/cByJ variants in Gdi1 and Irs4 discovered in this study do not seem to be relevant to CM ploidy, it is possible that these variants may contribute to other phenotypic effects. The PolyPhen-2 prediction for the BALB/cByJ Irs4 Leu683Phe variant is “probably damaging” (score 0.998). The protein encoded by this gene is named as an insulin receptor substrate, and while it not clear that this protein is actually a substrate for the insulin receptor^16^, it is thought to participate in signaling processes that control metabolism^17,18^. In humans, mutations in IRS4 are associated with central hypothyroidism, although mice carrying an Irs4 null allele had unchanged serum thyroid hormone concentrations^19^. It remains unknown if the BALB/cByJ allele has related or other effects. Gdi1 encodes a Rab GDP dissociation inhibitor, which is involved in vesicular trafficking. In humans, GDI1 loss-of-function gene mutations are associated with X-linked intellectual disability (mental retardation)^20^, and in mice, deletion of Gdi1 results in memory and behavioral alterations that resemble the human condition^21^. The BALB/cByJ variant (Arg276Cys) has the potential to compromise protein function (PolyPhen-2 prediction of “probably damaging”, score of 0.994). Interestingly, Gdi1 deletion in mice is associated with less aggressive behavior^21^, and BALB/cByJ mice are reported to be less aggressive than BALB/cJ mice^22^, which would be consistent with the natural BALB/cByJ variant identified here having functional consequences.

## Methods

### Animals

All mice in this study were obtained from The Jackson Laboratories (BALB/cJ JAX #000651; BALB/cByJ JAX #001026) or were bred in-house from these stocks. All mouse analyses were performed on mice 8–10 weeks of age. Animals were euthanized by isoflurane anesthesia followed by cervical dislocation and removal of hearts. Animal research was reviewed and approved by the IACUC committees of the Univ. of Southern California (#10173) and of the Medical Univ. of South Carolina (2018–00642), and all experiments were performed in accordance with relevant guidelines and regulations.

### Single-cell ventricular cardiomyocyte suspensions and nuclear ploidy analysis

Following a methodology we have used previously^10^, hearts were digested *ex vivo* with 1 mg/ml collagenase type II in calcium-free Tyrode’s solution (120 mM NaCl, 4 mM KCl, 0.33 mM NaH_2_PO_4_, 1 mM MgCl_2_, 10 mM HEPES, 11 mM glucose, 20 mM taurine, 20 mM BDM) via Langendorff retroaortic perfusion. After digestion, atria and valves were removed and ventricular tissue alone was triturated in Kruftbrühe (KB) solution (70 mM potassium aspartate, 40 mM KCl, 15 mM KH_2_PO_4_, 10 mM glucose, 10 mM taurine, 0.5 mM EGTA, 10 mM sodium pyruvate, 10 mM HEPES, 5 mM BDM, 0.5% BSA), filtered by gravity through a 250μ nylon mesh, stained with LiveDead Fixable (ThermoFisher, L10120) in PBS for 20 min at room temperature and then fixed in 2% paraformaldehyde (PFA) in PBS at room temperature for 15 min. Fixed ventricular cell suspensions were stained for cTnT (1:1,000, Abcam ab8295) overnight at 4 °C followed by goat anti-mouse secondary (1:500, ThermoFisher A11001), washed with PBS, and resuspended in PBS containing 5 µg/ml DAPI for 5 min with rocking. Cell suspensions were washed once in PBS then pipetted across a slide and coverslipped. Numbers of nuclei per cardiomyocyte were quantified using photographs taken at a uniform setting for all cell preparations with a Leica DFC3000G camera in full frame mode (1296 × 966 pixels; 3.75µ^2^ pixel size) through an Olympus BX41 fluorescence microscope (20x objective). Only live cardiomyocytes were counted; at least 300 cells were counted per heart. An unpaired, two-tailed Student t-test was used to assess statistical significance when only two groups were compared. To evaluate the ploidy of CM nuclei, using ImageJ software, nuclei in photographs were identified and outlined with a standard threshold requirement for all samples, and DAPI fluorescence intensity of each nucleus automatically quantified by ImageJ. The median value of DAPI fluorescence intensity of CD31 + endothelial cell nuclei was used as a diploid nucleus standard and given a value of 1, all other nuclear fluorescence signals were normalized to this value. Nuclei were assigned as being diploid if their intensity value was within the 0.5–1.5 range (indicated by a red box in some figures), tetraploid for values 1.5–2.5, and octaploid for values>3.

### Hepatocyte isolation and ploidy analysis

After severing the portal vein, mouse livers were perfused via a 24 gauge needle placed in the inferior vena cava with prewarmed 37 °C perfusion buffer (0.14 M NaCl, 6.7 mM KCl, 10 mM HEPES pH = 7.4, 0.1 mM EGTA) for 5–10 min at 7 ml/min until the liver was pale. The buffer was then changed to digestion solution (66.7 mM NaCl, 6.7 mM KCl, 100 mM HEPES pH = 7.4, 4.7 mM CaCl_2_, 1 mg/ml collagenase type II) and perfusion was continued for approximately 15 min at 3 ml/min. The liver was transferred to a petri dish containing ice-cold DMEM, and after the gallbladder was removed, was minced with forceps. The cell suspension was pipetted several times then filtered by gravity through 70 µ nylon mesh into a 50 ml tube. Cells were centrifuged at 210 × g for 3 min, resuspended in 5 ml 0.05% trypsin in PBS with 1 mM EDTA and incubated at 37 °C for 10 min with rocking, then centrifuged at 210 x g for 3 min. Cells were washed with PBS three times then fixed in 70% ethanol for 15 min. 10 µl of the fixed cell suspension was pipetted onto a glass microscope slide and air-dried. Slides were blocked with 10% normal goat serum (Thermo Fisher Scientific 50062Z) with 0.1% Triton-X100 for 1 h, then incubated with primary antibodies anti-CD31 (1:250, BD Pharmingen 553370) and anti-albumin (1:250,GeneTex GTX102419) at 4 °C overnight, followed by secondary antibodies Alexa Fluor 488 (Invitrogen A11001) and Alexa Fluor 546 (Invitrogen A10040) and with DAPI using standard procedures. Slides were coverslipped with ProLong Gold antifade reagent (Invitrogen) and photographed under fluorescence microscopy. Hepatocyte nuclei were identified and their fluorescence intensity quantified as performed for cardiomyocytes; roughly 200 hepatocytes were analyzed for each sample.

### Bone marrow cells isolation and ploidy analysis

Trimmed femurs from euthanized mice were flushed three times with 0.5 ml PBS using a 1-ml insulin syringe with a 29 gauge needle. The collected cells were transferred into a 1.5 ml eppendorf tube and fixed by adding 0.5 ml of 4% PFA in PBS (final PFA concentration 2%) and incubated at room temperature for 10 min. Cells were centrifuged at 100 x g rpm for 3 min, washed three times with PBS, and stained with DAPI using standard procedures. Photography and nuclear fluorescence intensity were as for cardiomyocytes; roughly 400 bone marrow cells were analyzed for each sample.

### Exome analysis and validation

BALB/cJ (6 independent samples) and BALB/cByJ (5 samples) exome sequence data were obtained from a previous analysis^23^ and from the Mouse Mutant Resource variant database (MMRdb: https://mmrdb.jax.org/mmr/). Using tools available in MMRdb and SAMTools (http://samtools.github.io/bcftools/bcftools.html), annotated variant calls in the variant caller format (VCF) from these samples were filtered to remove variants with rsID numbers, low quality variants (QUAL < 70), heterozygous variants (GT = 0/1), and autosomal variants. Calls that were shared between BALB/cJ and BALB/cByJ or shared with at least several other inbred strains were likewise removed. The remaining variants were novel, homozygous variant calls from the X chromosome. Gene fragments were amplified from mouse genomic DNA by PCR with GoTaq Green Master Mix (Promega); primers used are listed in Supplementary Table S1. Sanger sequencing of amplified fragments was performed by GeneWiz (genewiz.com) using one of the amplification primers.

### PolyPhen-2 SNP assessment

An on-line tool at http://genetics.bwh.harvard.edu/pph2/ was used with the human GDI1 (P31150) or IRS4 (O14654) Uniprot entry. For mouse Gdi1, the R276 position is the same in the human sequence. For mouse Irs4, the L883 position corresponds to position 711 of Uniprot O14654.

## Supplementary information

Supplementary information.

## Data Availability

All experimental data generated or analyzed during this study are included in this article and its Supplementary Information files.
