# Supplementary material for: Allelic variants between mouse substrains BALB/cJ and BALB/cByJ influence mononuclear cardiomyocyte composition and cardiomyocyte nuclear ploidy
Source: Sci Rep. 2020 May 5;10:7605. doi: 10.1038/s41598-020-64621-0 (PMC7200697; doi:10.1038/s41598-020-64621-0)
Supplement: Supplementary file 1 — Supplementary information. [file 41598_2020_64621_MOESM1_ESM.pdf]

Supplementary Information

Allelic variants between the mouse substrains BALB/cJ and BALB/cByJ influence mononuclear cardiomyocyte composition and cardiomyocyte nuclear ploidy

Peiheng Gan[1,2], Michaela Patterson[3], Hirofumi Watanabe[1], Kristy Wang[1], Reilly A. Edmonds[2], Laura G. Reinholdt[4], Henry M. Sucov[1,5,*]

[1]Dept. of Regenerative Medicine and Cell Biology, Medical University of South Carolina, Charleston, SC.
[2]Dept. of Stem Cell Biology and Regenerative Medicine, University of Southern California Keck School of Medicine, Los Angeles, CA.
[3]Dept. of Cell Biology, Neurobiology and Anatomy, and Cardiovascular Center, Medical College of Wisconsin, Milwaukee, WI.
[4]The Jackson Laboratory for Mammalian Genetics, Bar Harbor, ME.
[5]Dept. of Medicine Div. of Cardiology, Medical University of South Carolina, Charleston, SC.

5 Supplementary figures
1 Supplementary table

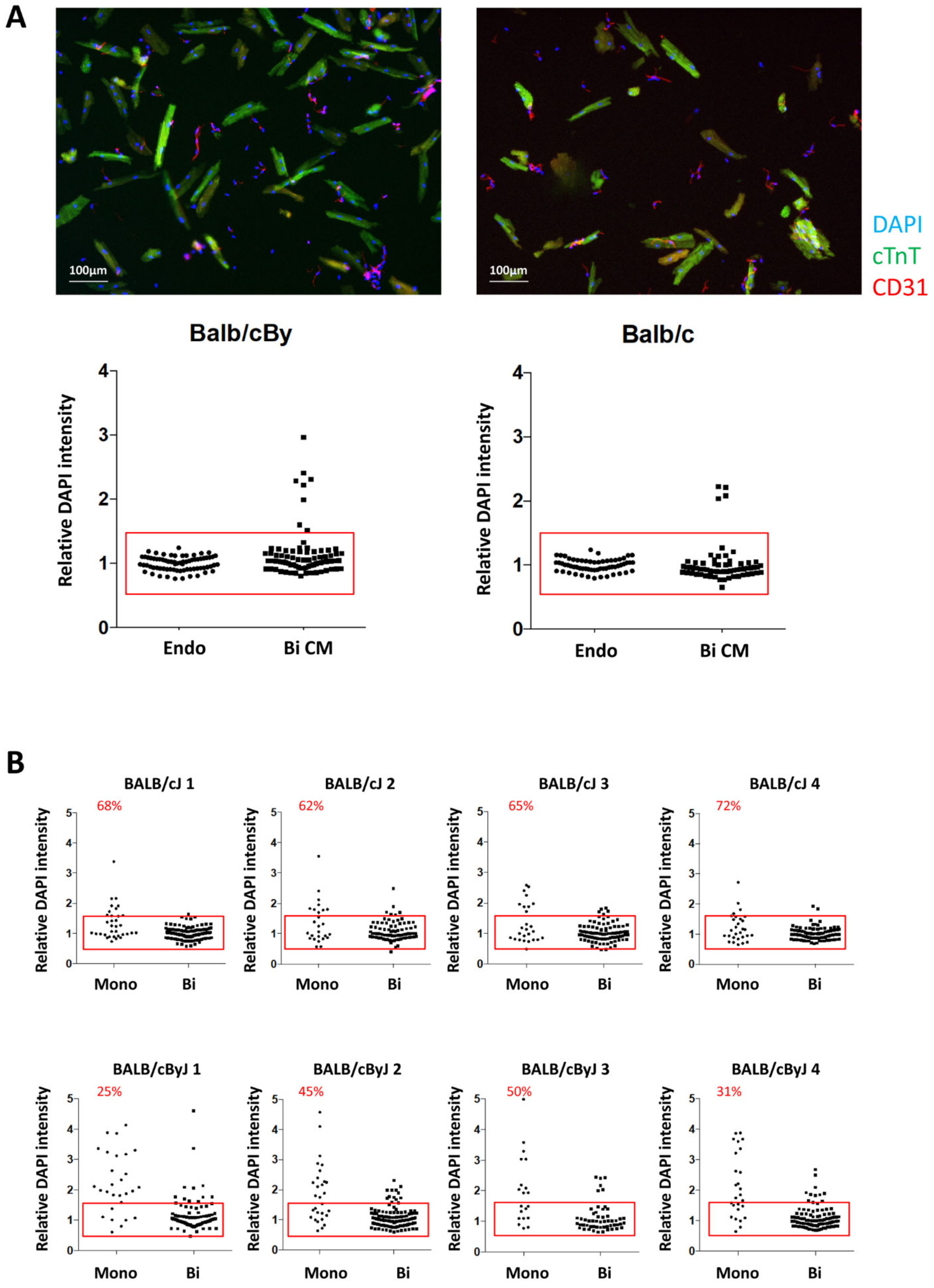
A
100µm
100µm
DAPI
cTnT
CD31
Balb/cBy
Balb/c
Relative DAPI intensity
Endo
Bi CM
B
BALB/cJ 1
68%
BALB/cJ 2
62%
BALB/cJ 3
65%
BALB/cJ 4
72%
BALB/cByJ 1
25%
BALB/cByJ 2
45%
BALB/cByJ 3
50%
BALB/cByJ 4
31%
Relative DAPI intensity
Mono
Bi

Supplementary Fig. S1. Quantitation of CM nuclear ploidy in parental strains. **A.** An example of using DAPI nuclear fluorescence of CD31+ endothelial cells as a reference for diploid nuclei, compared to nuclei from binucleated CMs. Cardiac troponin T (cTnT) is a CM-specific marker. In the charts, each dot represents the fluorescence intensity of a single nucleus in cells of the indicated type (Endo, endothelial cells; Bi CM, binucleated cardiomyocytes). All fluorescence intensity values are normalized to the median value of the endothelial cell nuclei population. The red box indicates the 0.5-1.5 threshold for inclusion of a nucleus as diploid. **B.** Primary data of four BALB/cJ and four BALB/cByJ hearts; each graph represents ventricular cardiomyocytes of a different animal. None of these were from the mice shown in panel A. The numerical values shown indicate the percentage of diploid nuclei specifically within the mononuclear CM population; these data points are graphed in Fig. 1B.

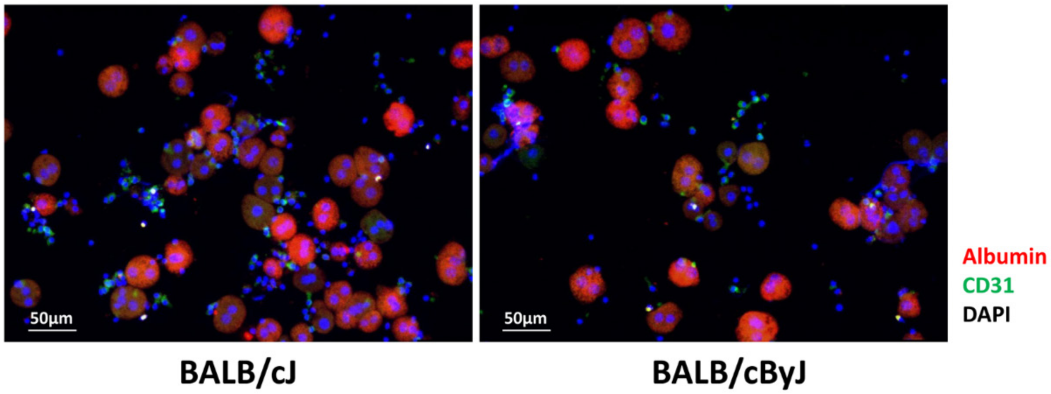

Supplementary Fig. S2. Representative images of isolated liver cells; hepatocytes are large and round after isolation and stain positive for albumin (red), many examples of mononuclear and binucleated hepatocytes are evident.

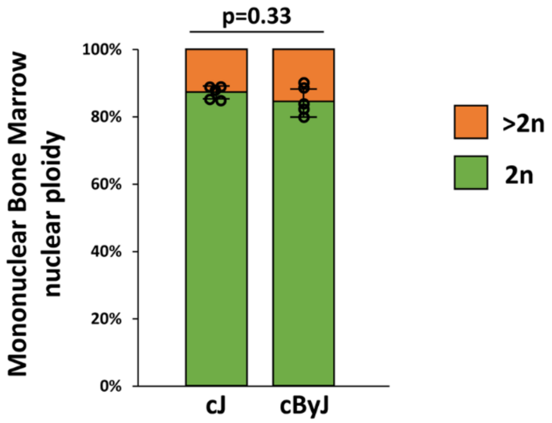

Supplementary Fig. S3. Evaluation of bone marrow cell polyploidy. All cell nuclei were from mononuclear cells; no binucleated cells were observed.

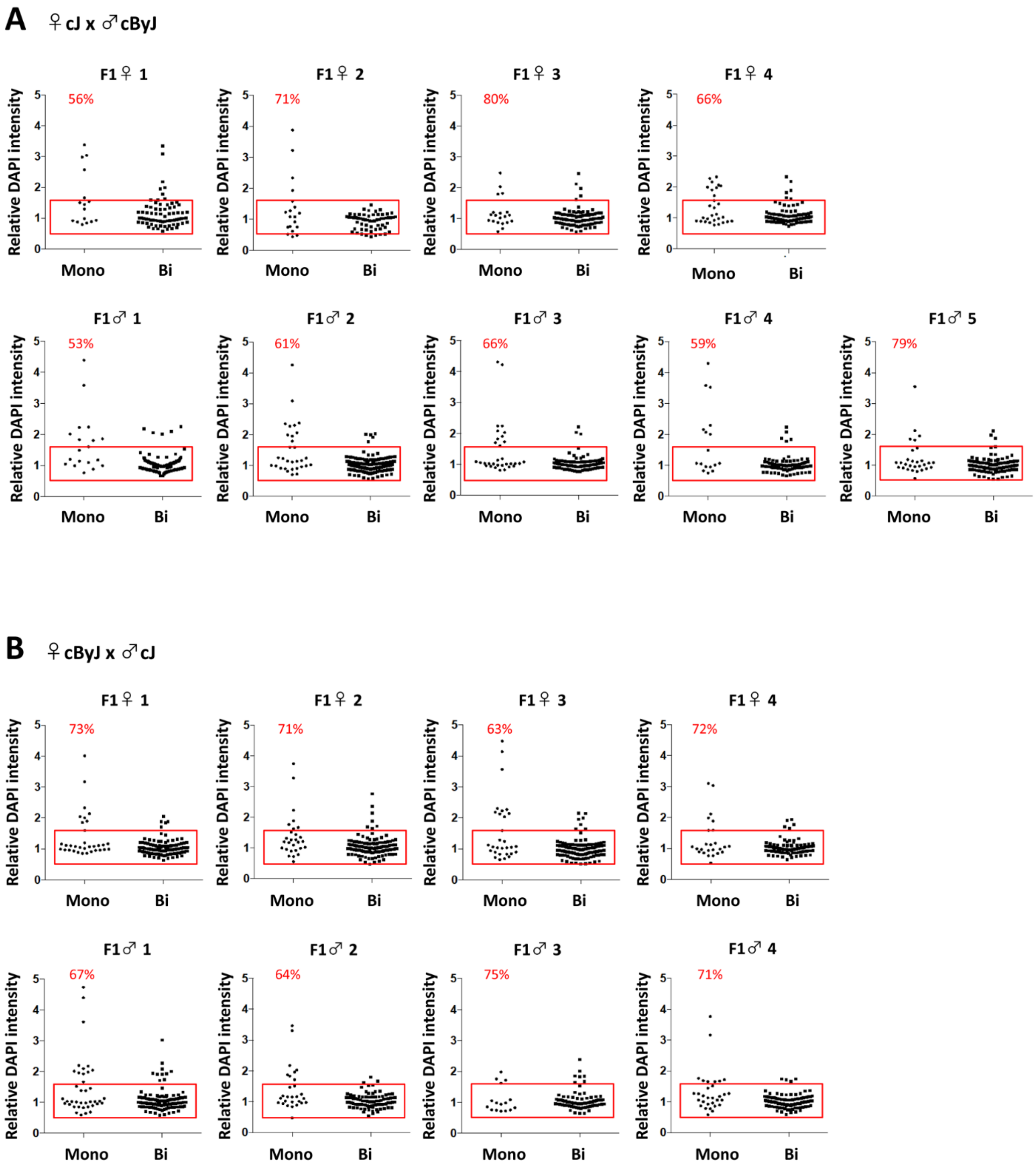

Supplementary Fig. S4. Quantitation of CM nuclear ploidy in F1 mice. **A.** Primary data for five male and four female F1 mice derived from crosses of BALB/cJ females to BALB/cByJ males. **B.** Primary data for four male and four female F1 mice derived from crosses of BALB/cByJ females to BALB/cJ males. The numerical quantitation values shown indicate the percentage of diploid nuclei specifically within the mononuclear CM population, and are graphed as data points in Fig. 1F.

Chr location GRCm38 mm10	dbSNP RS number	dbSNP 142 functional annotation More info	BALB/cJ	SEA/GnJ	C57BL/6J	129P2/OlaHsd	129S1/SvImJ	129S5SvEv<Brd>	AKR/J	A/J	BTBR T<+> Itp...	BUB/BnJ	C3H/HeH	C3H/HeJ	C57BL/10J	C57BL/6NJ	C57BR/cdJ	C57L/J	C58/J	CAST/EiJ	CBA/J	DBA/1J	DBA/2J	FVB/NJ	I/LnJ	KK/HlJ	LEWES/EiJ	LP/J	MOLF/EiJ	NOD/ShiLtJ	NZB/BlNJ	NZO/HlLtJ	NZW/LacJ	PWK/PhJ	RF/J	SPRET/EiJ	ST/bJ	WSB/EiJ	ZALENDE/EiJ
X:57092486	rs31755951 A/G	Cn:Vgll1:GR:72	A	A	G	G	G	G	G	A	G	G	A	A	G	G	G	G	G	G	G	G	G	G	G	G	G	G	G	G	G	G	G	G	G	G	G	G	G
X:161192220	rs255759965 C/T	Cn:Scml2:LP:145	C	C	T	T	T	T	T	T	T	T	T	T	T	T	T	T	T	C	T	T	T	T	T	T	C	T	C	C	T	T	T	C	T	C	T	C	C
X:161842605	rs29303490 C/T	Cn:Nhs:VI:583	T	T	C	C	C	C	C	C	C	C	C	C	C	C	C	C	C	T	C	C	C	C	C	C	T	C	T	C	C	C	C	T	C	T	C	T	C
X:163936672	rs213006026 A/G	Cn:Zrsr2:YH:415	G	G	A	A	A	A	G	A	A	A	A	A	A	A	A	A	A	G	A	A	A	A	A	A	G	A	G	A	A	A	A	G	G	G	A	G	G
X:163979223	rs31413241 C/G	Cn:Car5b:PA:308 I:LOC102641516	C	C	G	G	G	G	C	G	G	G	G	G	G	G	G	G	G	G	G	G	G	G	G	G		G	C	G	G	G	G	C	C	C	G	C	G

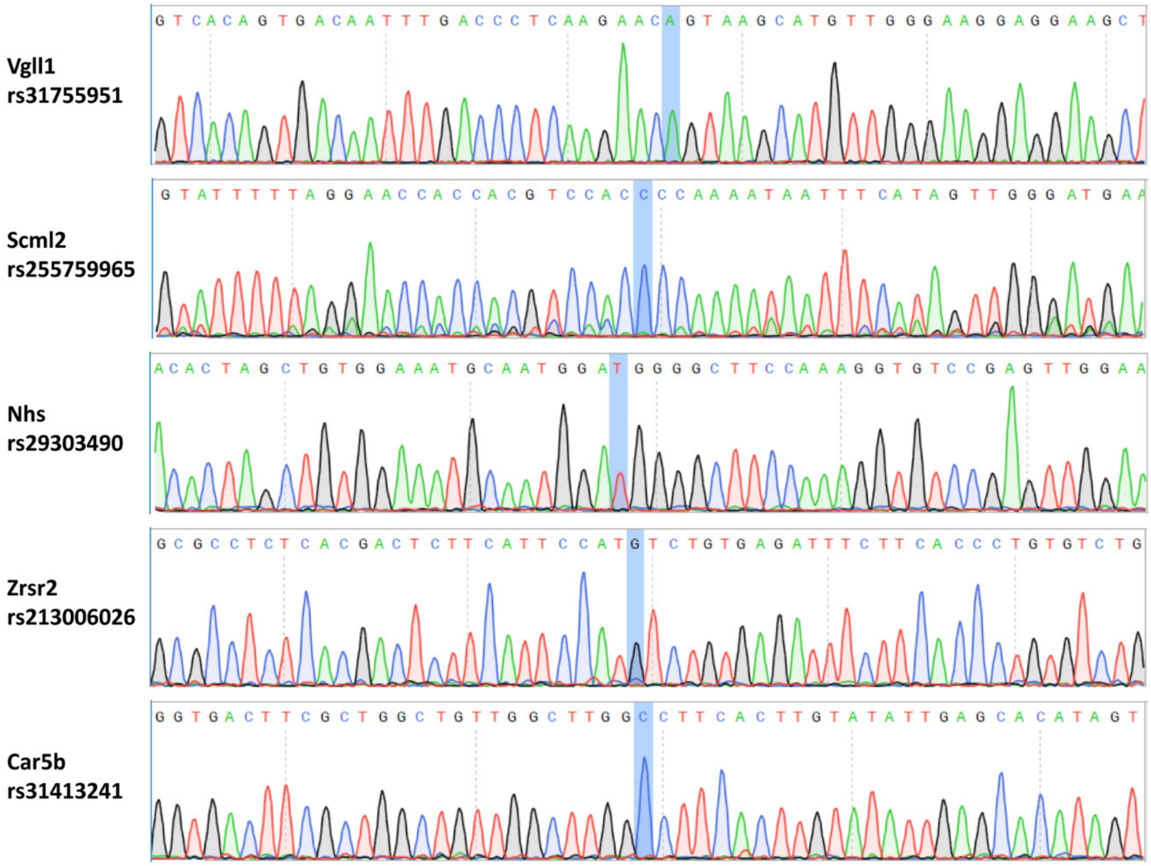

Supplementary Fig. S5. BALB/cJ variants found to not be polymorphic with BALB/cByJ. The table shows a screenshot from the phenome.jax.org web tool of selected SNPs identified in BALB/cJ based on comparison to all available mouse whole genome sequence. SEA/GnJ was derived in part from BALB/cJ and so is grouped with BALB/cJ in this table. The sequence traces show genomic sequencing of these candidate SNPs in BALB/cByJ; all SNPs are identical in BALB/cJ and BALB/cByJ.

Supplementary Table 1. Sequences of primers used for gene amplification and sequencing

Gene	Forward primer	Reverse primer
Vgll1	ACCTGAAAGCAGTCAAAAACCG	AGAGCAGCAAACTCAGCCTT
Scml2	CCATGACACCTGGCCTACAAA	ACATCTCCTGTGAGGCGACA
Nhs	TGTTGTTGGGGTCATCCAGC	TCCAAGCAGCCCAAGTACAC
Zrsr2	GCCACGACTTCTGGACCTTG	GGACTAGCTCCTCCTTCGGTA
Car5b	TTGTCTCCCGGTCTTTGCTTT	GTTGGAAGCCAACCTGGTCTA
Gdi1	TGTGATCATTGTGTTTTAGCAGTAGGG	CCCCAACCTCCCCATAAAGTTT
Irs4	CCTTTATTGGTGTCCGGTGCT	AGACCTGGAGATGGTCATGGC
